# Nutritional Quality and Popability of Popcorn (*Zea mays* L. var. everta) in Response to Compost and NPK 20-7-3 Application under Dryland Condition of South Africa

**DOI:** 10.1155/2023/6115098

**Published:** 2023-06-15

**Authors:** Oyeyemi A. Dada, Sydney Mavengahama, Funso R. Kutu

**Affiliations:** ^1^Food Security and Safety Niche Area Research Group, North-West University, Mafikeng Campus, Mmabatho 2735, South Africa; ^2^Department of Crop Protection and Environmental Biology, University of Ibadan, Nigeria; ^3^School of Agricultural Sciences, University of Mpumalanga, Mbombela 1200, South Africa

## Abstract

The dietary value of popcorn, an important snack, depends on its proximate and nutritional constituents, while the economic worth is based on popability and expansion traits of the kernels. There is paucity of information on how soil fertility influences or relates with popping potentials as well as quality of popcorn kernel in semi-arid region. Therefore, the proximate composition and popping parameters of popcorn in response to organic and inorganic fertilizers were investigated. The field trial was conducted in 2017-2019, and it comprised five amendment rates including 90 and 180 kg ha^−1^ NPK fertilizer and 4 and 8 t ha^−1^ compost and unamended treatment as the control. The trial was arranged in randomized complete block design in triplicate. Data on kernel yield, biomass, and harvest index were evaluated. Kernels were analysed for proximate composition and popping indices using standard procedures. Across the two seasons, mean protein (8.1%) and fibre (10.2%) contents were highest in kernels from plots fertilized with NPK at 180 kg ha^−1^, while grains from plots fertilized with 8 t ha^−1^ compost had the highest moisture (19.3%) and starch (50.1%) contents. The highest kernel expansion of 54.18 cm^3^ g^−1^ and 77.6% popped kernels were obtained in plots fertilized with 4 t ha^−1^ compost. Most of the kernels (61%) were small-sized caryopsis. Popability is significantly associated with volume expansion (*r* = 0.696). Proximate components and popability improved greatly in compost-augmented field relative to the unfertilized plots. Application of 4 or 8 t ha^−1^ sorted municipal solid waste compost to Luvisol enhanced growth and nutritional quality of popcorn. In view of promoting nutrient cycling towards improving soil fertility without compromising environmental health, compost is comparable and a good alternative to fossil-based mineral fertilizers.

## 1. Introduction

The quality of marketable yield obtainable from a particular crop species is influenced by myriads of intrinsic and extrinsic factors [1]. Such factors as nutritional and organoleptic appeal have been identified as determinants of popcorn (*Zea mays* L. var. *everta*) quality [2]. Kernel expansion volume plays a vital role in the economic value, consumers' preference, and acceptability of popcorn. One of the major intrinsic factors is kernel-related traits such as size, weight, colour, type, and proximate composition [3]. The quality of popcorn is not only affected by these factors but by other inherent qualitative characteristic of seeds that contribute significantly to the quality of popcorn kernels according to Ahmet and Kapar [4]. Popability is a major qualitative trait in popcorn for both commercial purpose and consumer preference. This is because popcorn cultivars with high popping percentage are highly patronized by growers for high returns on investment. On the other hand, Karababa [5] has shown that consumers relish popcorn with high organoleptic value and overall acceptability based on the popped kernel shape, volume, palatability, and colour. Arnhold et al. [6] and Jele et al. [7] identified popping ability as heritable quality. This probably explains why high popping ability is significantly related to kernel size and yield per unit area [8]. In addition, the qualitative determination of macromolecule constituents and the extent of kernel expansion are major criteria for determining popcorn quality [9]. Mass and expansion volume of kernels are important traits that enhance commercial value of popcorn [10]. In South Africa, there is dearth of information on popcorn quality, seed-related traits, and genotype influence on the quality of popcorn [7].

Edaphic factor like soil nutrient sources has influence on the final quality obtained from popped corn [8], but information is scanty on how improving soil fertility is associated with popping indices. Many researches have reported on improvement in the quality and popping traits of popcorn from Northern and Southern America with sketchy information from many African countries where popcorn breeding programs are fairly prioritized [7, 11].

In South Africa, popcorn is a major snack in many recreational centers with estimated annual consumption worth of US $12.26 million in 2017 and expected to increase to $15,098 million by 2023 at approximately 7% growth rate according to Thakur et al. [12] and Serna-Saldivar [13]. South Africa has favourable environment and potentials for popcorn cultivation that could meet its market demand [14, 15]. However, there is currently a huge shortfall in production, leading to massive importation from other countries especially America [15, 16]. More than 95 percent of popcorn kernels used in the country are imported from overseas according to Jele et al. [7]. Worse still, only a small proportion of the local consumption is harvested from fairly more than 2000 ha to complement unpopped kernels imported from North America, Europe, and Asia Pacific [12]. Only pockets of outgrowers in few of these cultivable lands are dedicated to popcorn production [14].

Organic-based fertilizers such as compost, farm yard manure, and green manure application have been identified as affordable and good sources of mineral nutrients for crop growth and development [17]. The constituents of compost have been shown to enhance soil physical and chemical properties better than synthetic fertilizer sources perhaps because of their organic components [18] as well as residual effects that reduce the cost expend on annual fertilizer application [18, 19]. It was observed that the skeletal strength of macroaggregates of intensively cultivated Vertisol improved tremendously, while enzyme activities on the macroaggregates were also enhanced when organic fertilizer is applied [20]. The quality of the final harvestable product is directly related to the quality of the soil, regarding plant nutrients and their sources. According to Berkhout et al. [21], the quality of harvested produce for human consumption can be improved by enriching marginal soils with amendments that are rich in the deficient minerals as well as organic matters.

There are copious reports on improvement in crop productivity as response to compost application. Apparently, how this translates to nutritional quality has not been sufficiently explained because field trials are rare in this direction [22]. Besides, empirical data on the optimum organic or inorganic fertilizer rates require to achieve improvement in popcorn quality especially in semi-arid regions where growth is scanty. Most growers treat popcorn the same way as other maize types in terms of agronomic practices. The objective of this study was to investigate proximate composition and popping attributes of popcorn in response to co-composted mixture of animal with sorted municipal solid wastes and inorganic NPK 20-7-3 fertilizer on loamy soil under dryland condition of South Africa. The outcome is expected to be disseminated to popcorn growers in the region so as to increase popcorn production.

## 2. Materials and Methods

A 2-year agronomic field trial was conducted during 2017/2018 and 2018/2019 summer plant seasons at the North-West University (NWU) experimental farm, Mafikeng Campus (25°49′39^″^S, 25°36′3^″^E, and 1280 masl). The soil of the study area was locally classified as Hutton [23] and Ferric Luvisol by Fey [24]. The site had been previously used for researches on legumes such as cowpea, dry bean, and cereal crops like sorghum and maize. During this trial, no other maize type was planted around the vicinity of the study site. The mean precipitation and temperature for 2017/2018 summer planting season were 646.40 mm and 28°C, respectively, which were higher than 428.01 mm and 23.10°C, respectively, during 2018/2019 summer planting season.

### 2.1. Compost Preparation

The compost was prepared from sorted municipal solid waste (MSW) at the composting site of the NWU experimental farm. Co-composting of sorted MSW with poultry manure (PM), cattle manure (CM), and sheep manure (SM) was undertaken to improve the quality of the MSW compost. The PM, CM, and SM were collected from the Animal Science Unit of NWU experimental farm, while MSW was collected from a nearby waste dumping site. The MSW was sorted to remove nondegradable materials. A heap containing a mixture of the organic materials was prepared at a ratio of 5 : 4 : 2 : 3 (MSW:PM:CM:SM) on dry weight (kg) basis. The chemical composition of the sorted municipal solid waste prior to cocomposting is as reported by Dada and Kutu [25]. Thermophilic anaerobic heap method described by Kutu et al. [26] was used for the decomposition process. Layers of the materials were made according to the ratio on flat floor laid with 100 mm thick plastic sheet to form a heap under a shed. The heap was turned biweekly until maturity, and the entire composting period lasted for four months. Precomposting analysis of nitrogen, phosphorus, potassium, and carbon in each of the raw materials as well as the final cocomposted product is shown in Table 1.

### 2.2. Field Establishment and Management

The field was ploughed and harrowed two weeks later, after which treatments were laid out on the field in a randomized complete block design with three replicates. There were five treatments comprising two rates, each of NPK 20-7-3 fertilizer (90 and 180 kg ha^−1^) and compost (4 and 8 t ha^−1^) with unamended plots included to serve as the control. There was no rate recommended for popcorn in South Africa at the time of this study; hence, the upper and lower levels of mineral fertilizers and compost usually recommended for growing *Zea mays* in the region were evaluated.

One popcorn seed was sown per hole of 3 cm depth in 3 m × 2.1 m plot size at a spacing of 20 m × 70 cm (20 cm intrarow and 70 cm interrow) to obtain a population of 101,578 plants ha^−1^. Mineral fertilizer was applied at planting, while the cured compost was applied three weeks prior to sowing to allow for adequate mineralization being an organic material that requires further degradation before making its nutrients available for crop use. All other agronomic practices were applied as recommended for *Zea mays* cultivation in dryland environment [27]. The plots were weeded manually at 3 and 6 weeks after sowing. Although the experiment was rainfed, supplementary irrigation was applied as required and established through visual observation on grown plant. Overhead sprinkler irrigation was adapted across the entire field. Each block was stationed with two sprinklers which were operated to irrigate the field every other day depending on soil moisture level. Stem borer infestation was managed in the field by spraying beta-cyfluthrin (Bulldock 25SC) at the rate of 400 mL ha^−1^. The ears were allowed to mature and dry on the field before harvesting at less than 15% grain moisture content.

### 2.3. Data Collection and Measurements

At harvest, data were collected on kernel yield, biomass, and harvest index. Kernels were analysed for popping indices and proximate analysis following standard methods described by Ahmet and Kapar [4] and Jele et al. [7].

### 2.4. Determination of Popping Potential (Popability) Traits

Popping potential of the corn in response to the treatments was carried out at the analytical science laboratory of Animal Science Department at NWU using modified hot air popping method described by Jele et al. [7]. Popping was done in triplicate for each treatment. The popping process was standardized by popping some trial samples on hot air stove (intermediate high at 800 W power) before popping the treatment samples. Samples of popcorn kernels (50 g) were measured into a hot stainless medium size pot (20 cm × 9.7 cm) containing 10 mL oil and placed on electric stove (Samsung PKG, 500/FA) used as the heat source. The pots with the kernels were shaken on the stove continuously for two to three minutes after which popping was completed.

The moisture content of grains harvested from each plot was measured using the Dole® moisture tester. Kernel size was determined by measuring 10 g samples and then counting the number of kernels per 10 g as described by Allred-Coyle et al. [28]. The kernels were classified into small (76-105), medium (68-75), and large (52-67) following the procedures of Song and Eckhoff [29] and Karababa [5] for categorizing popcorn kernels into different sizes. Flake volume was measured using a 1000 cm^3^ measuring cylinder tapped once to settle popcorn flakes. The percentage of unpopped kernels was determined and recorded for each sample. Popped popcorn samples were poured into 1000 cm^3^ measuring cylinder and inverted once to record the volume, while volume of unpopped kernels was evaluated. Popping volume was evaluated as

(1) $$\begin{array}{c} \text{Popping volume}=\frac{\text{ total popped volume }\left({\text{cm}}^{3}\right)\;}{\text{original sample weight }\left(\text{g}\right)}, \end{array}$$

while unpopped kernel was evaluated in percentage as

(2) $$\begin{array}{c} \text{Percentage of unpopped kernels}=\frac{\text{number of unpopped kernels}}{\text{total number of kernels}}\times 100, \end{array}$$

according to Sweley et al. [30].

The flake size was estimated as

(3) $$\begin{array}{c} \text{Flake volume}=\frac{\text{ total popped volume }\left({\text{cm}}^{3}\right)\;}{\text{number of popped kernels}}. \end{array}$$

### 2.5. Proximate Analysis of Popcorn Kernels

The proximate mineral compositions of the kernels were determined in triplicate following standard procedures and using NIR spectrometer (Unity Scientific 2500XL SpectraStar NIR Spectrometer). The machine was calibrated for popcorn, and 50 g kernel sample was measured from each treatment. The 50 g kernel sample was poured into scanning cup and then scanned to read the proximate contents.

### 2.6. Statistical Analysis of Data

Analysis of variance (ANOVA) was done on data collected using GLM of statistical analysis system [31]. Variation in means was delineated with LSD at *p* ≤ 0.05. Association among the popping variables and kernel indices was also evaluated with the Pearson product moment correlation (PPMC) at *p* ≤ 0.05.

## 3. Results

Different rates of soil amendment exerted significant (*p* < 0.01) effect on all the proximate contents of popcorn kernels except polyunsaturated fatty acid (PUFA) in the first season, while sugar, nitrogen detergent fibre (NDF), and acid detergent fibre (ADF) were not significantly affected by the different soil amendments in both cropping seasons (Table 2). During 2017/2018 season, grain yield, kernel size, and total biomass were highest in plots treated with 8 t ha^−1^ compost relative to the unamended plot which had the lowest yield of 3.82 t ha^−1^, kernel size of 2.74 mm, and total biomass of 103.18 g (Table 3). In 2018/2019 season, grain yield was highest in plots supplied with 180 kg ha^−1^ NPK but was statistically comparable to the yield obtained in plots fertilized with 90 kg ha^−1^ NPK. The kernel size (2.92 mm) and total biomass (168.33 g) were highest in plot treated with 8 t ha^−1^ compost but were statistically similar to all other treatments. The harvest index of 1.10 and 1.59 was highest in plots fertilized with 90 kg ha^−1^ NPK in 2017/2018 and 2018/2019 seasons, respectively, but comparable to harvest index obtained from plot supplied with 8 t ha^−1^ compost. The unfertilized plots had the lowest harvest index in both experimental periods.

The response of nutritional constituents of popcorn kernel to different rates of organic and inorganic soil amendments as reflected by the proximate composition during 2017/2018 and 2018/2019 seasons is presented in Table 4. The moisture content was highest in plots fertilized with 8 t ha^−1^ compost but not significantly different from moisture content recorded in plot amended with 4 t ha^−1^ compost. Kernels from unfertilized plots had lowest moisture contents. Similar trend was observed for starch content in both seasons. Polyunsaturated and monounsaturated fatty acids were highest in plots amended with 90 kg ha^−1^ NPK but statistically similar to polyunsaturated and monounsaturated fatty acids obtained in plots amended with 8 t ha^−1^ compost. Kernels obtained from the plots fertilized with 4 t ha^−1^ compost had highest protein composition (8.3%) in 2017/2018 season, while plots fertilized with 90 kg ha^−1^ NPK had kernels with highest protein (8.4%) in 2018/2019 season. Protein content in both seasons was lowest in kernels from unamended plots. The fibre content of 10.1 and 10.3% in 2017/2018 and 2018/2019 seasons, respectively, was highest in kernel harvested from plots fertilized with 180 kg ha^−1^ NPK and statistically similar to fibre content in kernel from plots amended with 90 kg ha^−1^ NPK. The lowest fibre contents of 8.0% and 8.2% for both planting seasons were recorded in kernels from plot fertilized with 8 t ha^−1^ compost. The highest ash content of 3.7 and 3.8% was obtained in kernels from plots fertilized with 180 kg ha^−1^ NPK in 2017/2018 and 2018/2019 seasons, respectively, while the lowest ash content of 3.0% for both planting seasons was recorded in kernels from unamended plots. Kernels harvested from the plots fertilized with 4 t ha^−1^ had highest starch content, while the kernels from the unamended field had the lowest starch (44.4 and 43.5%) in both seasons. Sugar concentration in the kernels of popcorn differed significantly with the highest sugar obtained in kernels obtained from unamended plots and lowest from kernels fertilized with 8 t ha^−1^ compost (Table 4). The NDF and ADF compositions of the kernels were not affected by different rates of soil amendments.

Different rates of soil amendments influenced popping indices of popcorn kernels as indicated in Table 5. The mean of proximate composition across the two seasons showed that kernels harvested from compost-fertilized plots had higher flakes, popped volume (expansion), popped percentage, and length of kernel and lesser unpopped percentage than the constituents obtained from NPK-fertilized plots. Application of compost at the rate of 4 t ha^−1^ increased the volume of flake (270.92 cm^3^ g^−1^) and expansion (54.18 cm^3^ g^−1^) significantly compared to the same attributes in kernel obtained from plot fertilized with 90 kg ha^−1^ NPK which had the lowest. Highest popped percentage of 77.6% and lowest unpopped kernel of 54.7% were recorded in kernels from plot fertilized with compost at rate of 4 t ha^−1^, but these were not significantly different from results obtained in plot fertilized with 8 t ha^−1^ compost.

The length of kernel was highest in plot amended with 8 t ha^−1^ compost which was not significantly different from length of kernels obtained from plot supplied with 4 t ha^−1^ compost. Wider kernel was obtained from field augmented with 180 kg ha^−1^ NPK, and this was statistically similar to the results obtained in the plots augmented with 90 kg ha^−1^ NPK (Table 5). Kernel breadth of 0.87 mm was highest in popcorn obtained from plot fertilized with 180 kg NPK. Also, the mean number of kernels per 10 g (102.50) was more in plots fertilized with 180 kg ha^−1^ NPK. However, mass of ten kernels was not statistically influenced by the different rates of soil amendments as indicated in Table 5.

**Figure 1:**
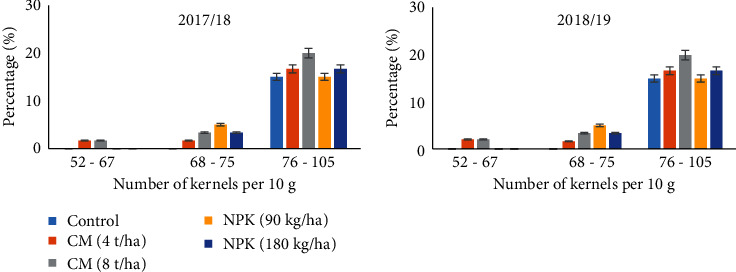
Distribution of popcorn kernel sizes in response to organic and inorganic fertilizers during 2017/2018 and 2018/2019 seasons.

The distribution of kernel based on size as influenced by different soil amendments is shown in Figure 1. Majority of the kernels in a 10 g sample were grains with small size; only few were large grains. In both seasons, plots fertilized with 8 t ha^−1^ compost had kernels with the smallest size, while medium-sized kernels were recorded in field augmented with 90 kg ha^−1^ NPK (Figure 1).

Association among popping traits of popcorn kernels is shown in Table 6. Flake volume has significant and direct association with total volume, popped volume, percentage popability, and length of kernel. However, unpopped kernels were negatively correlated with total volume, popped volume, and length of kernel. Breadth of kernel was negatively associated with total volume. Popped volume was negatively associated with unpopped kernel, breadth of kernel, and number of kernels in 50 g sample.

## 4. Discussion

Response of popcorn to mineral fertilizer is comparable to that of organic amendments in terms of kernel quality as well as grain expansion volume. The improved performance recorded in popcorn regarding yield and its attributes in response to compost-fertilized plots suggests that physical and chemical properties of marginal soil could be greatly enhanced by mineral constituents in the applied compost. The importance of compost as a source of organic minerals in improving the fertility of nutrient-deficient soils has been widely reported by Kirchmann et al. [32] and Adekiya et al. [33]. Our study confirmed the outcome of previous studies regarding effects of compost on barley [34], wheat [35], and other cereals [36]. Compost and other organic fertilizers are good sources of available and highly mineralized organic materials that could be reused or recycled to minimize environmental pollution, while encouraging greener ecosystem and economy [37, 38]. Since the effect of organic fertilizers is akin to fossil-based mineral fertilizers, gradual reduction in mineral fertilizer usage could be incorporated into extant farming system in order to reduce the rate of carbon emission and ensure cleaner environment [32, 39]. Hence, efforts should be geared towards extensive incorporating compost application in fertilization program of cereal crops as a mean of enhancing food security and reducing the negative effects of fossil-based synthetic fertilizers and its resultant impacts on climate change [40, 41].

The decline in grain yield during the second planting season may be attributed to variation in agroclimatological or environmental variables especially reduction in the amount of precipitation during 2018/2019 season compared to 2017/2018 cropping season. Similar reason was provided for variation in yield of rice by Sridevi and Chellamuthu [42] as well as for oilseed rape by Botai et al. [43]. It is evidently clear that climate change may have adverse effect on yield and yield indices of cereal crops in this region. The better yield recorded under compost application during the first trial may be connected to better mineralization due to adequate soil moisture level relative to the second season when soil moisture level might be low due to reduced precipitation. Hence, the need to factor effect of unpredictable change in climate into arable cropping system in order to prevent food insecurity cannot be overemphasized.

The comparable harvest index (HI) of organic amendments to inorganic fertilizer explained high nutrient use efficiency of the applied amendments for production of high economic yield relative to biological components. This underscores the fact that the applied fertilizers were highly mineralized such that the uptake was efficiently metabolized into kernel yield better than development of morphological attributes. Similar view had been shared by Uwah et al. [44] on sweet maize and Bekeko [45] on hybrid corn in eastern Ethiopia.

The higher percent of starch and sugar contents in kernels obtained from compost-fertilized plots may be attributed to organic matter component which possibly enhanced synthesis of some complex organic compounds in the photoassimilates [46]. Also, Naveed et al. [47] have reported that application of organic fertilizers such as composted chicken manure and other farmyard manures induced bioavailability of micronutrients in harvestable produce.

Soil amendments that are high in nitrogen content have been reported to improve protein content in grains of some cereal crops as nitrogen is a building framework for amino acids and protein in many yield components according to Kumar et al. [48]. Although the protein contents in kernels obtained from either compost- or NPK-fertilized plots are comparable, the values were quantitatively higher in NPK-fertilized plots. This could be linked to the quality of nitrogen content in either of the amendments [49]. The higher protein content recorded in kernels from NPK-fertilized plots in comparison to kernels harvested from compost plots may mean that nitrogen from compost was not readily available to plant due to slow mineralization, hence resulting in the lower protein content. Lester and Saftner [50] have reported that the rates of nitrogen delivery influence the quality of protein content in crops. Our observation that mineral fertilizer promoted protein synthesis better than compost contradicted the report of Loria et al. [51] on effects of swine manure on corn protein content. Our view however agrees with the report of Nelson and Motavalli [52] that sources of mineral nutrients especially the essential elements are necessary determinant of proximate constituents of corn.

Mineral nutrient source is a major determinant of macromolecule contents in grains. This probably explains why fibre and fat contents were higher in kernels harvested from plots treated with inorganic fertilizer. In Sudan, Amin [53] and Osuagwu and Edeoga [54] have reported similar observations in corn and scent leaves, respectively, where they showed that fibre, ash, and protein contents varied in response to nitrogen sources. The rates of organic and inorganic fertilizer applications play significant role in proximate composition of popcorn kernels.

The ash content which is a measure of inorganic mineral contents explains the nutritional quality of the popcorn. The increase in ash content with increase in the amount of the amendments implies the suitability of either soil amendments in improving mineral content of the kernel. The poor performance recorded in unfertilized field is a clear indication that nutritional quality of popcorn will be adversely affected when grown on marginal soil without fertilizer application. Other proximate components were better in the amended plots compared to the unamended (control) plots. Abdullah [55] has also reported that popcorn is a rich source of snack which is low in energy and has high excellent fibre and minerals.

Kernel volume and expansion are desirable quality traits in popcorn because of their economic importance. Flakes and expansion volume have been linked to better soil fertility according to Ahmet and Kapar [4] and Singh et al. [56]. The higher kernel flakes and volume observed in compost-fertilized plots supposed that organic fertilizer source could be a better option for improving the fertility of popcorn field and ultimately kernel expansion indices. Sweley et al. [30] and Zulkadir et al. [57] have reported similar observations in different popcorn cultivars in Turkey and India, respectively. The higher quantity of popped kernel recorded in kernels obtained from organic fertilizer plots could be connected to the amount of moisture content and the kernel size as explained by Singh et al. [56]. The range of moisture content in the kernel falls within the limit when optimum expansion volume could occur according to Sweley et al. [2, 55].

Small- and medium-sized caryopses are more preferred for commercial purposes than those with large kernels. This is because kernels of small and medium sizes show greater flake and expansion volume than the larger kernel sizes [29, 30]. The percentage of unpopped kernel in the compost-fertilized plots was within the acceptable limit from oil-popped kernels as reported by Sona et al. [58] and Sweley et al. [2]. The number and size of kernel are related to improvement in soil fertility by the applied materials, which varied with different amendments, but the indices were superior to the kernel obtained from the unfertilized plots. Pordesimo et al. [59] and Bayomy [60] noticed similar response in physical and popping characteristic of yellow and purple popcorn kernels. The need to improve kernel traits with popping characteristics is very important since this is a major determinant of returns on investment in popcorn production. Therefore, the association between expansion traits and kernel size cannot be undermined. Besides, the range of unpopped characteristics can be reduced by selecting kernel with narrow breadth and better popping volume.

## 5. Conclusion

Popcorn nutritional values, expansion, and popping traits improved tremendously on Ferric Luvisol fertilized with sorted municipal solid waste compost, and the effect was comparable to inorganic fertilizer. The proximate constituent of popcorn which is the measure of popcorn quality was superior in compost-augmented field relative to the unfertilized plots. Hence, utilization of compost derived from solid waste materials is a good source of plant nutrients, means of nutrient recycling and mitigation of solid waste based environmental pollution. Application of 4 to 8 t ha^−1^ properly prepared municipal solid waste compost to Ferric Luvisol is beneficial to popcorn, a crop of high economic and dietary importance in dry land region of South Africa. Further study into establishing the optimum compost rate and the relationship between precipitation and moisture holding potential of compost as a mean of enhancing crop water use efficiency under limited water condition are expedient to maximize popcorn production in semiarid region.

## Figures and Tables

**Table 1:** Nutrient contents in the soil, compost components, and the final compost.

Mineral nutrient content	Soil	Manure			MSWC^∗^
		Sheep	Cattle	Poultry	
Total N (g kg^−1^)	0.52	1.96	2.23	2.48	35.56
Total P (g kg^−1^)	0.11	9.89	8.56	14.10	1.42
K (g kg^−1^)	0.33	2.42	77.54	39.20	10.22
Organic C (g kg^−1^)	17.85	30.2	28.5	28.5	133.56

^∗^Municipal solid waste compost product after the cocomposting process.

**Table 2:** Mean squares, coefficient of variation (CV (%)), and means of proximate characteristics of popcorn kernel.

Sources of variation	MC	PUFA	MUFA	Protein	Ash	Fibre	Starch	Sugar	NDF	ADF
	2017/2018 cropping season									
Rates of soil amendments	2.16^∗^	2.36	42.89^∗∗^	1.66	0.86^∗∗^	7.74^∗∗^	20.65^∗^	4.97	9.03	10.52
Mean	4.40	5.36	16.20	7.77	3.38	9.31	47.50	2.81	27.10	16.96
CV (%)	17.05	14.80	20.04	11.99	13.72	14.99	9.57	70.24	41.97	25.15
Pr > F	0.02	0.01	0.008	0.13	0.009	0.009	0.06	0.30	0.99	0.68
	2018/2019 cropping season									
Rates of soil amendments	2.06^∗^	2.12^∗^	50.84^∗∗^	1.92	1.18^∗∗^	8.93^∗∗^	78.48^∗^	8.73	166.44	14.02
Mean	4.74	5.37	16.33	7.75	3.40	9.31	47.76	2.77	27.91	17.13
CV (%)	19.04	16.23	19.92	13.68	17.17	14.44	11.77	72.85	39.37	26.70
Pr > F	0.04	0.03	0.000	0.14	0.01	0.000	0.04	0.07	0.24	0.64

Significant at ^∗^*p* < 0.05 and significant at ^∗∗^*p* < 0.001. MC = moisture content; NDF = neutral detergent fibre; ADF = acid detergent fibre; PUFA = polyunsaturated fatty acids; MUFA = monounsaturated fatty acids.

**Table 3:** Yield and yield components of popcorn in response to different rates of organic and inorganic soil amendments.

Soil amendment	Rate	2017/2018 cropping season				2018/2019 cropping season			
		GY (t ha^−1^)	Kernel size (mm)	TBM (g plant^−1^)	HI	GY (t ha^−1^)	Kernel size (mm)	TBM (g plant^−1^)	HI
Control	0	3.82	2.74	103.18	0.65	3.02	2.63	129.79	0.41
Compost (t ha^−1^)	4	6.03	2.83	105.23	0.93	3.11	2.83	139.54	0.48
	8	7.18	2.75	125.58	0.94	3.10	2.92	168.33	0.74
NPK 20-7-3 (kg ha^−1^)	90	6.79	2.83	108.71	1.10	5.45	2.91	99.19	1.59
	180	6.26	2.75	111.66	0.89	5.74	2.93	164.22	1.00
LSD (*p* ≤ 0.05)		2.41	0.39	40.72	0.44	1.83	0.38	75.94	0.89

GY = grain yield; TBM = total biomass; HI = harvest index.

**Table 4:** Proximate composition of popcorn kernels as affected by different rates of soil amendments.

Soil amendment	Rate	Proximate constituent (%)									
		MC	PUFA	MUFA	PROT	Fibre	Ash	Starch	Sugar	NDF	ADF
	2018/2019 cropping season										
Control	0	13.5	4.8	5.3	7.1	10.0	3.0	44.4	3.9	26.1	15.5
Compost (t ha^−1^)	4	17.7	4.2	4.7	8.3	8.6	3.1	50.3	2.5	27.8	17.4
	8	19.3	4.7	5.5	7.7	8.0	3.6	49.8	1.8	26.8	18.5
NPK 20-7-3 (kg ha^−1^)	90	15.0	5.6	6.2	7.6	10.0	3.6	47.6	3.2	26.3	16.2
	180	15.4	4.4	5.1	8.1	10.1	3.7	45.5	2.6	28.6	17.3
LSD (*p* ≤ 0.05)		3.3	0.8	0.8	1.0	1.4	0.5	4.6	2.0	11.6	4.3
	2018/2019 cropping season										
Control	0	13.9	4.8	4.8	7.2	8.5	3.0	43.5	2.1	23.4	16.7
Compost (t ha^−1^)	4	17.3	4.2	5.4	7.5	9.0	3.1	50.3	2.2	31.0	18.0
	8	19.2	5.0	5.5	7.4	8.2	3.3	50.3	2.1	24.4	16.9
NPK 20-7-3 (kg ha^−1^)	90	14.9	5.3	6.0	8.4	10.0	3.6	48.5	4.0	27.5	15.6
	180	14.9	4.4	5.1	8.0	10.3	3.8	47.5	2.6	33.1	18.7
LSD (*p* ≤ 0.05)		2.7	0.7	0.7	0.9	1.1	0.5	4.6	2.3	9.0	3.7

MC = moisture content; PROT = protein; NDF = neutral detergent fibre; ADF = acid detergent fibre; PUFA = polyunsaturated fatty acids; MUFA = monounsaturated fatty acids.

**Table 5:** Popping indices of popcorn kernels as influenced by organic or inorganic soil amendments.

Soil amendment	Rate	Volume (cm^3^/g)		Percentage (%)		Kernel		Number kernel (10 g)	Mass (g) 10 kernels
		Flake	Expansion	Popped kernel	Unpopped kernel	Length (mm)	Breadth (mm)		
Control	0	69.92	13.98	24.1	232.1	0.55	0.62	83.16	1.30
Compost (t ha^−1^)	4	270.92	54.18	77.6	54.7	0.84	0.60	84.25	1.28
	8	265.13	53.03	74.7	55.3	0.86	0.61	87.75	1.23
NPK 20-7-3 (kg ha^−1^)	90	52.29	10.46	36.7	359.0	0.56	0.83	86.17	1.42
	180	57.17	11.48	38.7	333.1	0.59	0.87	105.50	1.38
LSD (*p* ≤ 0.05)		38.58	8.76	24.5	122.7	0.07	2.00	16.85	0.21

**Table 6:** Correlation among popping indices of popcorn kernels as influenced by organic or inorganic soil amendments.

	Flake volume	Unpopped kernels	Total volume	Popped volume	Popability (%)	Unpopped kernel (%)	Breadth of kernel	Length of kernel	No. of kernel/50 g
Flake volume	1	-0.584^∗∗^	0.986^∗∗^	0.986^∗∗^	0.696^∗∗^	-0.398^∗∗^	-0.685^∗∗^	0.697^∗∗^	-0.081
Unpopped kernels		1	-0.692^∗∗^	-0.692^∗∗^	0.031	0.933^∗∗^	0.528^∗∗^	-0.567^∗∗^	0.242^∗^
Total volume			1	1.000^∗∗^	0.610^∗∗^	-0.513^∗∗^	-0.722^∗∗^	0.726^∗∗^	-0.125
Popped volume				1	0.610^∗∗^	-0.513^∗∗^	-0.722^∗∗^	0.726^∗∗^	-0.125
Popability (%)					1	0.292^∗^	-0.426^∗∗^	0.579^∗∗^	0.041
Unpopped (%) kernel						1	0.360^∗∗^	-0.398^∗∗^	0.186
Breadth of kernel							1	-0.450^∗∗^	0.273^∗^
Length of kernel								1	-0.087
No. of kernel/50 g									1

^∗∗^Correlation is significant at *p* < 0.01 level. ^∗^Correlation is significant at *p* < 0.05 level.

## Data Availability

The set of data used in this study has not been published elsewhere either partly or in its entirety. The data sets used and/or analysed during the current study are available from the corresponding author on request.
